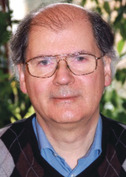# Reagents for Selective Fluoromethylation: A Challenge in Organofluorine Chemistry

**DOI:** 10.1002/anie.201913175

**Published:** 2020-06-04

**Authors:** Marco Reichel, Konstantin Karaghiosoff

**Affiliations:** ^1^ Department of Chemistry Ludwig-Maximilian University Butenandstr. 5–13 81377 Munich Germany

**Keywords:** electrophiles, fluoromethylating agents, monofluoromethylation, nucleophiles, radical reactions

## Abstract

The introduction of a monofluoromethyl moiety has undoubtedly become a very important area of research in recent years. Owing to the beneficial properties of organofluorine compounds, such as their metabolic stability, the incorporation of the CH_2_F group as a bioisosteric substitute for various functional groups is an attractive strategy for the discovery of new pharmaceuticals. Furthermore, the monofluoromethyl unit is also widely used in agrochemistry, in pharmaceutical chemistry, and in fine chemicals. The problems associated with climate change and the growing need for environmentally friendly industrial processes mean that alternatives to the frequently used CFC and HFBC fluoromethylating agents (CH_2_FCl and CH_2_FBr) are urgently needed and also required by the Montreal Protocol. This has recently prompted many researchers to develop alternative fluoromethylation agents. This Minireview summarizes both the classical and new generation of fluoromethylating agents. Reagents that act via electrophilic, nucleophilic, and radical pathways are discussed, in addition to their precursors.

## Graphical abstract

**Reagents** that introduce the CH_2_F group into organic molecules operate via electrophilic, nucleophilic, or radical routes. New stable, easy to handle, and efficient reagents for fluoromethylation provide access to organic CH_2_F derivatives of academic interest and industrial importance. While CH_2_F transfer to heteroatoms is straightforward, carbon fluoromethylation still remains a challenge.

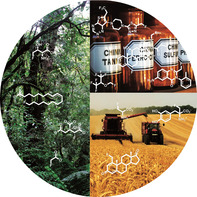

## 1 Introduction

### 1.1 General Overview

**Figure 1:**
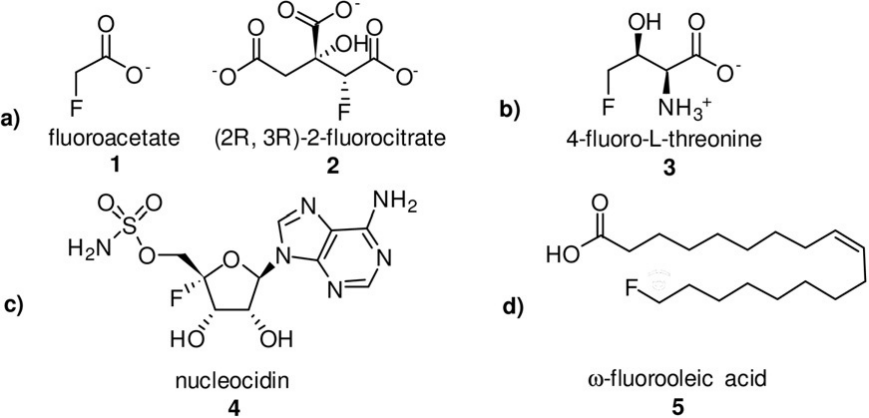
The five naturally occurring organofluorine compounds, which are found in plants, animals or bacteria; a) *Dichapetalum cymosum*, b) *Streptomyces cattleya*, c) *Streptomyces calvus*, d) *Dichapaetalum toxicarium*.

Fluorine occurs abundantly in nature as fluorspar and fluoroapatite.^1^ Despite these widespread natural resources, only one enzyme exists that has been confirmed to be able to perform fluorination: fluorinase. However, current research suggests that there might be at least one more enzyme capable of fluorination.^2^ Perhaps surprisingly, among an estimated number of 130 000 natural products, there are only five naturally occurring organofluorine compounds present in plants, bacteria, or animals (Figure 1).^1^, ^2^

**Figure 2:**
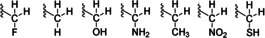
Selected functional groups to which the ‐CH_2_F moiety is bioisosteric.

Fluoroacetate is the most common of the naturally occurring organofluorine compounds and occurs in about 40, mostly poisonous plants in the southern and tropical regions of Africa, Australia, and Brazil.^2^, ^3^ Considering that organofluorine compounds are almost absent in nature, it is remarkable that 20 % of all pharmaceuticals and 30–40 % of all agrochemicals contain fluorine.^4^ The reason for this is simple and can be clearly illustrated by considering the toxicity of *Dichapetalum cymosum*. For fluoroacetate, the C−F bond prevents the conversion of this compound into cis aconitate, and stops at the 2‐fluorocitrate stage blocking the citrate cycle.3b Their metabolic stability and other unique physical, chemical, and biological properties of organofluorine compounds make them particularly interesting for the pharmaceutical and agricultural industries.^5^ These features make the monofluoromethyl group highly versatile as a bioisosteric unit for a series of functional groups found in biological systems (Figure 2).^6^

**Figure 3:**
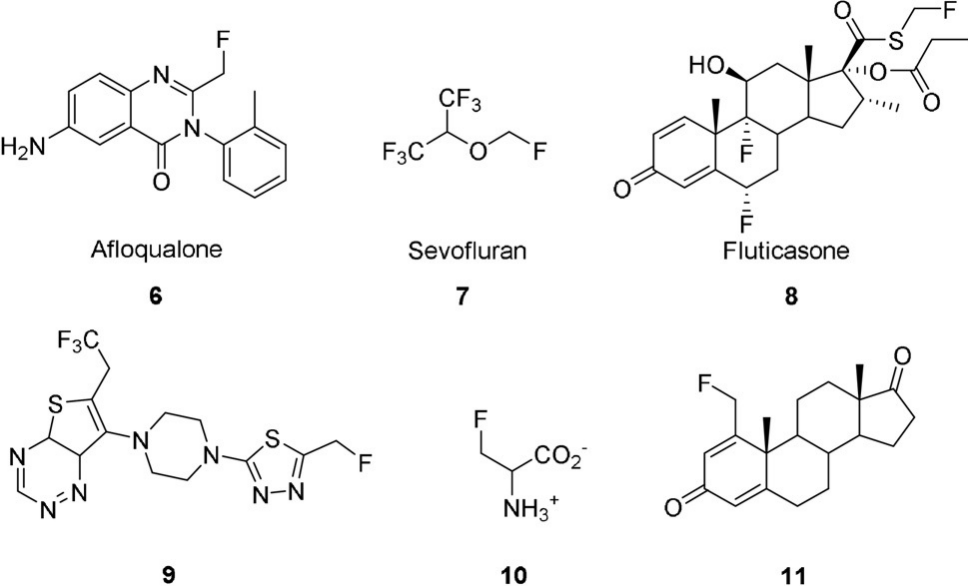
Selected drugs and inhibitors containing a fluoromethyl group.

This bioisosterism, combined with the enhanced metabolic stability, bioavailability, lipophilicity, and membrane permeability imparted by the fluorine substituent, allows for efficient drug design.^7^ As a result, a variety of monofluoromethylated drugs and inhibitors have been developed (Figure 3). For instance, Afloqualone (**6**) is a muscle relaxant and sedative with clinical use. Sevofluran (**7**) is a volatile anesthetic with great significance in pediatric anesthesia because of its good hypnotic but only weak analgesic and muscle‐relaxing properties. Fluticasone propionate^TM^ (**8**), a drug widely used against inflammatory diseases and as an analgesic in the treatment of certain cancers, is one of the industrially most important drugs.7b, 8 In addition to these well‐established drugs, a number of inhibitors have also been tested.6a, 9

Compound **9**, is an inhibitor for the tumor suppressor protein menin. The β‐fluorinated amino acid **10** acts as so‐called “suicide substrate”, which can deactivate decarboxylase enzymes and can be used against Parkinson's disease. The androsta‐1,4‐diene‐3,17‐dione **11** acts as an aromatase inhibitor and is suitable for the treatment of estrogen‐dependent diseases such as anovulatory infertility, prostate hyperplasia, breast cancer, and many more.6a, 9

**Figure 4:**
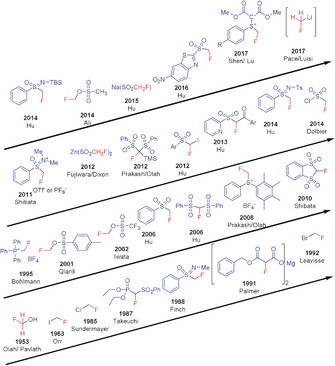
Historical overview of monofluoromethylating reagents and year of their first use as a CH_2_F transfer reagent.

The compounds CH_2_FBr (HFBKW‐31) and CH_2_FCl (HFCKW‐31) are frequently used on a large scale in industry for synthesis^10^ even though these compounds have high ozone‐depleting potentials.^11^ As these substances are going to be subject to successive banning under the Montreal Protocol, and the handling of these chemicals will have to follow increasingly stricter rules,11b alternative fluoromethylating agents are urgently needed. Although a fluoromethyl group can be generated by introducing fluorine in place of a suitable functional group^12^ or by direct monofluorination,^13^ the majority of synthetic procedures use a fluoromethylating agent instead, which can directly transfer a CH_2_F group.^14^ A further method starts with a precursor compound that formally transfers a “CFR_2_” unit (R=SO_2_Ar or others) to the substrate in the initial step, and subsequently gives the desired CH_2_F group after work‐up.7a Fluoromethylation chemistry developed before 2009 has been nicely reviewed by Hu and co‐workers.7a In addition, Review articles focusing on fluorine‐containing functional groups,5b difluoro‐ and fluoromethylation,^14^ transition‐metal‐mediated di‐ and monofluoroalkylations,^15^ sulfur‐based fluorination and fluoroalkylation reagents,^16^ and on shelf‐stable reagents for fluoro‐functionalization reactions^17^ have been published. This Minireview provides an overview over the reagents used for the specific introduction of the CH_2_F group into organic compounds. Classical monofluoromethylating agents as well as newly developed reagents have been considered (Figure 4). The literature has been covered until the end of 2019. The reagents were classified by considering their ability to either directly transfer the CH_2_F group in electrophilic, nucleophilic, or radical fluoromethylation reactions, or to act as suitable precursors generating CH_2_F after proper workup. The introduction of CH_2_F moieties by transition‐metal‐mediated cross‐coupling reactions is discussed in the Section covering the corresponding reagent.

### 1.2 A Historical Overview of Monofluoromethylating Reagents

The number of monofluoromethylating reagents has almost doubled over the last ten years (Figure 4), reflecting a dramatic development in this field. Particularly active in this area has been the group of Hu, providing eight of these reagents. Starting with simple compounds such as fluoromethanol and the fluoromethyl halides CH_2_FX (X=Cl, Br, I), more sophisticated and efficient reagents applicable to a broad range of substrates have been developed with time. Efforts were focused on the introduction of better leaving groups as compared to the halides and on fluoromethylating reagents acting as nucleophiles—the generation of CH_2_FLi being certainly a highlight—or reacting through a radical pathway. Over the last ten years, in particular reagents and synthetic protocols for radical fluoromethylation as well as for CH_2_F introduction through transition‐metal‐mediated cross‐coupling—mainly, but not exclusively, based on fluoromethyl halides—have been developed.

## 2 Reagents for Direct Monofluoromethylation

### 2.1 Electrophilic Monofluoromethylation

Fluoromethanol was the first reagent to be used for the electrophilic introduction of CH_2_F. Olah and Pavlath reported in 1953 the formation of fluoromethyl‐substituted arenes upon reaction with FCH_2_OH in the presence of a Lewis acid (ZnCl_2_).^18^ Recently, it has been used for the fluoromethylation of special alcohols.^19^

#### 2.1.1 Fluoromethyl Halides

**Figure 5:**
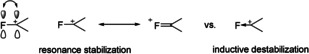
The α‐fluorine effect.

The fluoromethyl halides CH_2_FX (X=Cl, Br, I) are all volatile, which represents a challenge when using these compounds. Nonetheless, this property is also an advantage as this volatility allows for an excess of the reagent to be readily separated from the product. In general, CH_2_FX halides are weak fluoromethylating agents. Fluoromethylation through an S_N_2 reaction mechanism is more difficult than the analogous methylation with a methyl halide.5b, 20 The α‐fluorine effect is responsible for this behavior (Figure 5).^21^

**Figure 6:**
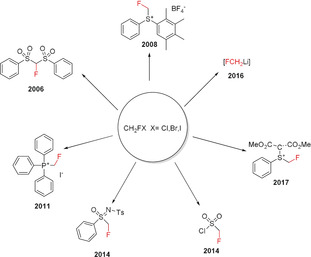
Selected strong fluoromethylating agents derived from fluoromethyl halides and year of their first application as a CH_2_F transfer reagent.

**Scheme 1:**
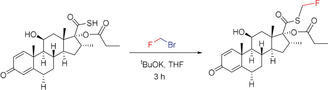
Fluoromethylation step in the synthesis of Fluticasone^TM^.

**Scheme 2:**
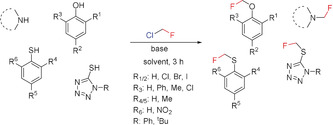
Fluoromethylation of various O, N, and S nucleophiles with CH_2_FCl.

A fluorine atom in the α‐position stabilizes a positive charge by π‐donation. This effect is so strong that the destabilizing inductive effect can effectively be ignored, and an S_N_2 reaction can only take place if a good leaving group is present at the CH_2_F moiety.21b, 21c Thus, the reactivity of the CH_2_FX halides increases in the order Cl<Br<I. However, some reactions such as the electrophilic fluoromethylation of carbon nucleophiles, as well as CH_2_F transfer to weak nucleophiles, are problematic.^22^ The fluoromethylating strength of CH_2_FX can be increased considerably through the presence of silver cations to bind the halide,21b, 23 making the fluoromethylation of weak nucleophiles such as NO_3_^− [23]^ and ClO_4_^− [21b]^ possible. Initially, CH_2_FI (Orr,^24^ 1963) and later CH_2_FBr (Lesuisse,^25^ 1992) and CH_2_FCl (Sundermeyer,^26^ 1985) was used for the fluoromethylation of a large number of substrates.^25^, ^26^, ^27^ The alkylation of a series of oxygen, sulfur, nitrogen, and carbon nucleophiles by fluoromethyl halides has been described.7a Moreover, fluoromethyl halides have often been used as starting materials for more efficient fluoromethylating agents (Figure 6).^28^ The first fluoromethylated compounds acting as aromatase inhibitors, or compounds with anabolic properties, were prepared using CH_2_FI and CH_2_FBr.^24^, ^25^ A series of ^18^F‐labeled fluoromethyl‐containing compounds that are frequently used for positron emission tomography (PET) imaging have been prepared by employing CH_2_^18^FBr.^29^ One of the most important applications of CH_2_FBr is its use in the last step of the synthesis of Fluticasone^TM^,^30^ which involves the fluoromethylation of a thiocarboxylate precursor at the sulfur atom (Scheme 1). Fluoroiodomethane^[27c–e, 31]]^ and the monosubstituted derivatives CHRFI27f, 27g and CHRFBr27f, 27g, 27h have been used in several cases to introduce a CH_2_F or CHRF group. The first systematic studies on the fluoromethylation of phenols, thiophenols, imidazoles, and indoles with CH_2_FCl (Scheme 2) were reported in 2007 by Hu and co‐workers.5b, 22

**Scheme 3:**
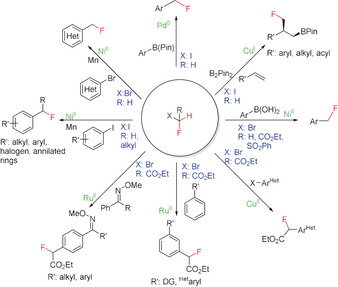
Transition‐metal‐mediated introduction of CH_2_F starting from fluoromethyl halides and monosubstituted derivatives.

Over the last ten years, several transition‐metal‐mediated fluoromethylation reactions starting from fluoromethyl halides CH_2_FX (X=Br, I) or carbon‐monosubstituted derivatives thereof have been developed (Scheme 3). All of these syntheses involve C−C bond formation. Thus aryl boronic esters or aryl boronic acids can be converted into the corresponding fluoromethyl derivatives by coupling with CH_2_FI, CH_2_FBr, or CHRFBr (R=CO_2_Et, SO_2_Ph) in Pd^0^ (Suzuki,27i Hu,27c Qing^32^) Cu^I^ (Qing27e), or Ni^II^ (Zhang,27b X.‐S. Wang27f) catalyzed reactions, respectively. Ni^II^ in combination with Mn has been used to promote the introduction of CH_2_F (X.‐S. Wang27a) and CHRF (R=alkyl; X.‐S. Wang27g) into heteroarenes and arenes starting from suitable heteroaryl bromides and aryl iodides by reductive cross‐coupling.

The CH(CO_2_Et)F group has been introduced in the *p*ara (Zhao^33^) or *meta* position (G.‐W. Wang,27l Ackermann27m) by Ru^II^‐catalyzed reactions of CH_2_F(CO_2_Et) with the corresponding methoxyphenyl ketoximes or monosubstituted arene derivatives, respectively. It has been shown by Wu and co‐workers27m that 8‐aminoquinolines react with CHF(CO_2_Et)Br in the presence of Cu^II^ and HP(O)(OMe)_2_ to give the corresponding CHF(CO_2_Et)‐substituted derivatives.

It is noteworthy that the known fluoromethyl pseudohalides CH_2_FX (X=CN,^34^ NCO,^35^ N_3_ 36) have not yet been used as fluoromethylating agents.

#### 2.1.2 Fluoromethyl Sulfonates

**Scheme 4:**
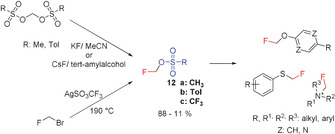
Alkylation with fluoromethyl sulfonates.

The fluoromethyl sulfonates **12 a** (Ali, 2014),^37^ **12 b** (Qianli, 2001),28l and **12 c** (Iwata, 2002)^38^ have been used to introduce CH_2_F into a series of compounds at oxygen, sulfur, or nitrogen atoms (Scheme 4).7a The main and most important application of these reagents is in the synthesis of ^18^F‐labeled fluoromethyl compounds to enable PET imaging.^39^ The fluoromethyl sulfonates **12 a** and **12 b** have been prepared starting from bis(mesyloxy) and bis(tosyloxy) methane and by introducing fluorine by reaction with KF.^40^ The synthesis of **12 b** has been considerably improved^41^ and is almost quantitative when CsF in *tert*‐amyl alcohol is used to introduce fluorine.^12^ Fluoromethyl triflate **12 c** has been obtained from CH_2_FBr and silver triflate,^38^, 39b albeit under quite harsh reaction conditions.28d Since 2009, the use of these reagents has greatly increased, and more non‐^18^F‐labeled compounds have been synthesized in a targeted manner.28h, 42

#### 2.1.3 *S*‐Monofluoromethyl Diarylsulfonium Tetrafluoroborate

**Scheme 5:**
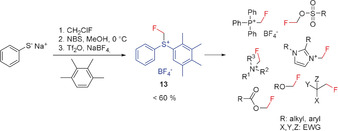
Fluoromethylation with **13**.

In 2008, Prakash and Olah developed a powerful fluoromethylating agent that has been successfully applied in the fluoromethylation of numerous nucleophiles (Scheme 5). The fluoromethylsulfonium salt **13** is obtained in a three‐step synthesis with an overall yield of 60 %.28b Interestingly, the first step—the synthesis of the fluoromethyl phenyl thioether—is reported with better yields in the literature.5b The sulfonium salt **13** is a moisture‐insensitive solid; it is stable for several months in the solid state and is also stable in acetonitrile solution. However, in DMF and THF, decomposition occurs.28b

Substrates that possess heteroatoms as nucleophilic centers are readily fluoromethylated upon reaction with **13**. In particular, fluoromethyl sulfonates can be prepared under mild conditions by using the sulfonium salt **13**. However, its application to carbon nucleophiles has thus far remained limited to only a few compounds.28b

#### 2.1.4 *N*,*N*‐(Dimethylamino)‐*S*‐phenyl‐*S*‐monofluoromethyl Phenyloxosulfonium Triflate

**Scheme 6:**
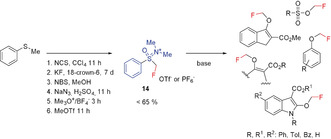
O‐Fluoromethylation of selected compounds.

A very effective fluoromethylating reagent was developed in 2011 by Shibata and co‐workers.^43^ It shows a pronounced preference for fluoroalkylation at oxygen atoms, which provides a synthetic approach for the preparation of monofluoromethyl ethers. This method was applied to a number of 1,3‐dicarbonyl compounds. It is a regioselective reagent for β‐keto esters and was successful also in the fluoromethylation of carboxylic and sulfonic acids, oxindole derivatives, and phenols, as well as naphthols (Scheme 6).^17^, ^43^

A disadvantage of this reagent is its tedious, multi‐step synthesis. However, when a modified procedure from the literature is used to simplify the synthesis of the fluoromethyl phenyl thioether intermediate,^44^ the overall synthesis time can be reduced substantially from almost 9 days to 1.5 days.28b, 43 The reagent **14** is a solid that is easy to handle and can be stored.^43^ Although O‐alkylation can also be performed well with other reagents, the *E*/*Z* stereoselectivity of **14** is particularly noteworthy. The O‐regiospecificity of **14** was explained by a radical‐like mechanism involving a SET process.^45^ However, Shen and co‐workers reported that alcohols did not react with this reagent under the conditions applied.28d

#### 2.1.5 Monofluoromethyl‐Substituted Sulfonium Ylides

Completing the series of difluoromethyl‐ and trifluoromethyl‐substituted sulfonium ylides, Shen and Lu reported in 2017 the missing monofluoromethyl sulfonium ylide **15**, which was structurally characterized by single‐crystal X‐ray diffraction. Reagent **15** is a stable solid and can be stored for at least one month at ambient temperature on the bench without notable decomposition, and it can be prepared in a straightforward manner in good yields.28d

**Scheme 7:**
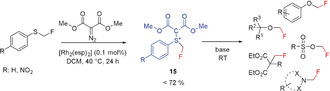
C‐, N‐, and O‐fluoromethylation with sulfonium ylide **15**.

The ylide **15** was found to be a very effective reagent for the electrophilic fluoromethylation of primary, secondary, and tertiary alcohols, as well as of malonic acid derivatives.28d It was shown that **15** is a strong alkylating agent. Thus, the conversion of sulfonic acids, carboxylic acids, phenols, amides, and N‐heteroarenes into the corresponding fluoromethyl derivatives takes place readily under mild conditions (Scheme 7).28d

Although **15** is a strong alkylating agent, reactions of **15** with carbon nucleophiles and C−CH_2_F bond formation are problematic, and only proceed with special substrates.28d

### 2.2 Nucleophilic Monofluoromethylation

**Scheme 8:**
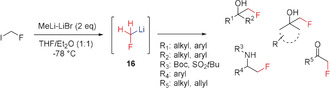
Nucleophilic fluoromethylation with fluoromethyl lithium **16**.

Because of their high instability, organometallic reagents such as fluoromethyllithium or the corresponding Grignard reagents belong to the most difficult areas of research on nucleophilic monofluoromethylating agents.7a In 2017, Pace and Luisi achieved a great breakthrough in this field. They reported the generation and use of fluoromethyllithium, which was the first and still remains the only direct nucleophilic monofluoromethylation reagent (Scheme 8).^46^ In order to perform reactions with this unstable species, it is important to stick strictly to the reaction conditions reported,^46^ as the generation of **16** only succeeds upon adding MeLi⋅LiBr in a molar ratio of 2:1.5 to the substrate. Furthermore, the reaction has to be quenched, and a solvent mixture of THF/Et_2_O (1:1) has to be used.^46^ Unfortunately, unlike MeLi, reagent **16** cannot be isolated at room temperature as decomposition occurs very quickly, most probably by elimination of LiF.

### 2.3 Radical Monofluoromethylation

#### 2.3.1 *N*‐Tosyl‐*S*‐fluoromethyl‐*S*‐phenylsulfoximine

**Scheme 9:**
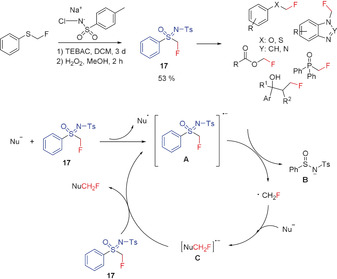
Radical fluoromethylation of selected O, S, N, P compounds with **17** and proposed reaction mechanism.

Until about ten years ago, a free radical monofluoromethylation was unknown.7a In 2014, Hu and co‐workers described the sulfur‐containing reagent **17**, which is able to transfer the fluoromethyl radical group to a substrate (Scheme 9).28e, 47

Various compounds were fluoromethylated at O, S, N, or P in good yields by using sulfoximine **17**. The range of applications of **17** was extended by Akita and co‐workers to the C‐fluoromethylation of alkenes by using strongly reducing photoredox catalysts.13a Despite the time‐consuming (3 days) synthesis of **17** and the only moderate yield, an important advantage of this reagent is its stability. At room temperature, **17** is a crystalline solid, which has been characterized by single‐crystal X‐ray diffraction and does not decompose even upon storage in air for one year.^47^

#### 2.3.2 Fluoromethylsulfonyl Chloride

**Scheme 10:**
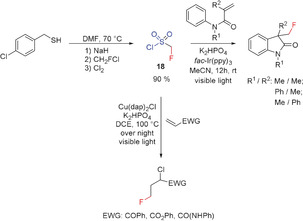
Radical fluoromethylation of *N*‐aryl acrylamides with **18**.

Concurrent with the development of sulfoximine **17**, in 2014, Dolbier and co‐workers developed a photoredox‐catalyzed tandem radical cyclization of *N*‐aryl acrylamides to form fluorinated 3,3‐disubstituted 2‐oxindoles using an iridium catalyst and fluoromethylsulfonyl chloride as the CH_2_F source (Scheme 10).28j

Sulfonyl chloride **18** (colorless oil) is readily obtained from 4‐chlorobenzyl thiol in three steps in excellent yield (90 %). In the cases of *N*‐phenyl acrylamide and electron‐deficient alkenes, instead of cyclization, a formal addition of chlorine and CH_2_F to the C=C double bond takes place to yield saturated derivatives with a terminal fluoromethyl group (Scheme 10). The reaction is catalyzed by copper and is induced by visible light. Both reactions also occur with CHF_2_ or CF_3_ substituents in place of CH_2_F.28c However, although the yields of the fluoroalkylated products are good, applications of this reagent still remain limited at the present time.

#### 2.3.3 Metal Fluoromethyl Sulfinates

**Scheme 11:**
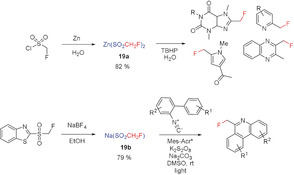
Free radical fluoromethylation of selected heterocycles.

In 2012, Fujiwara and Dixon described a radical fluoromethylation using the zinc fluoromethyl sulfinate **19 a**.^48^ This reagent enables the C−H functionalization of diverse heterocycles by introducing a fluoromethyl group (Scheme 11).

In 2015, Hu and co‐workers developed a large‐scale synthesis for sodium sulfinate **19 b** and used it for radical monofluoromethylation reactions.^49^ Later, in 2017, Liu and co‐workers demonstrated that sodium sulfinate **19 b** is a suitable reagent for the transition‐metal‐free radical fluoroalkylation of isocyanides to form phenanthridines.^50^ Coumarin derivatives with a CH_2_F group have been prepared very recently by Li and co‐workers starting from alkoxynates by a silver‐catalyzed cascade monofluoromethylation with **19 b**.^51^ The zinc sulfinate **19 a** has also been widely used for the synthesis of bioactive compounds,^48^ and is remarkable because of its simple and straightforward synthesis. Compound **19 a** has been isolated as a colorless solid and is stable at room temperature. However, the synthesis of the sodium salt, starting from a heteroaryl sulfone, is much simpler.^49^

#### 2.3.4 Monofluoromethyl Sulfones

**Scheme 12:**
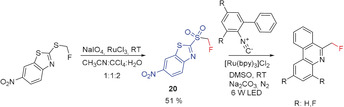
Metal‐mediated radical fluoromethylation of isocyanides.

In 2016, Hu and co‐workers reported a visible‐light‐induced photoredox synthesis of fluoromethyl‐substituted phenanthridines based on the reaction of suitable isocyanides with fluoromethyl sulfone **20**.^52^ The high redox potential of the fluoromethyl sulfone is essential for successful fluoromethylation, and an irradiation time of 48 h was required (Scheme 12).^12^, ^13^, ^14^, ^15^, ^17^, ^53^

The fluoromethylating reagent **20** is isolated in the last step in moderate yield as a colorless, air‐stable solid, which makes it easy to handle. Its overall synthesis, however, includes several steps and requires the use of CH_2_FCl as the source of the fluoromethyl group.^12^, ^13^, ^14^, ^15^, ^17^, ^53^

## 3 Indirect Monofluoromethylation

Because of the instability of organometallic fluoromethyl reagents such as fluoromethyllithium, it is sometimes necessary to use precursor compounds containing a functionalized fluoromethyl group. After the transfer of the functionalized group to the substrate, the desired ‐CH_2_F moiety is generated during workup.

### 3.1 Nucleophilic Precursors

#### 3.1.1 Fluoromalonates

**Scheme 13:**
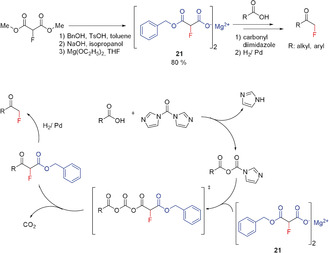
Synthesis of fluoromethyl ketones using magnesium fluoromalonate **21**.

In the 1980s, the monofluoromethylation of organic compounds attracted increasing interest. Research in this area was focused in particular on the development of mild fluoroalkylating reagents, complementing the traditional methods based on fluoromethyl halides. Palmer reported an effective alternative reagent for the fluoromethylation of carboxylic acids, namely the magnesium salt **21** (Scheme 13).7a, 54 The key step involves the nucleophilic attack of an intermediately generated fluoromethyl carbanion to the imidazolide of the carboxylic acid. Thus, reagent **21** may be viewed as a synthon of the unstable CH_2_F^−^ anion. The resulting β‐keto α‐fluoro esters give the corresponding fluoromethyl ketones upon hydrogenation in good yields. The starting fluoromalonate ester is readily prepared^54^, ^55^ and is nowadays commercially available. Fluoromalonate methyl55a and ethyl55b ester have also been directly used in fluoromethylation reactions. The formation of **21** (colorless solid) is straightforward, although it comprises three steps. Furthermore, despite intensive studies, it has not been possible to use this reagent in enantioselective transformations.7a

#### 3.1.2 Fluoromethyl Phenyl Sulfone and Related Compounds

**Scheme 14:**
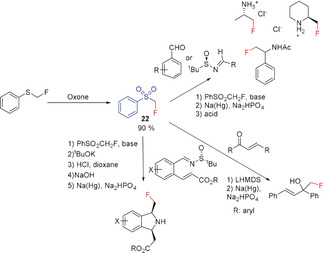
Fluoromethylation with fluoromethyl phenyl sulfone **22**.

Fluoromethyl phenyl sulfone (**22**) is a colorless solid that was reported as far back as 1985 to form the corresponding fluoromethylidene ylide, and it has been used to prepare fluoroolefins in a Wittig‐analogous reaction.^56^ In 2006, Hu and co‐workers extended this methodology to formally transfer the CH_2_F moiety, which is formed after cleavage of the sulfonyl group (Scheme 14).^57^ Thus starting from (*R*)‐(*tert*‐butylsulfinyl)imines, primary α‐fluoromethyl amines and cyclic secondary α‐fluoromethyl amines become readily accessible with high stereoselectivity using this reagent. The method was further extended by Fustero and co‐workers to include the synthesis of chiral fluoromethyl isoindolines^58^ and isoquinolines.^59^ Hu and co‐workers further successfully utilized **22** for the stereoselective synthesis of a vicinal fluoromethyl ethylene diamine.^60^ Monofluoromethyl‐containing amides can also be prepared using **22** in a Ritter reaction.^61^ The reaction of sulfone **22** with 2‐cyclohexanone and acyclic α,β‐unsaturated ketones proceeds both by addition to the carbonyl group as well as through a Michael addition, and yields the corresponding fluoromethyl derivatives after reductive cleavage of the sulfonyl group, as reported by Hu and co‐workers.7a, 58, 59, 62

**Scheme 15:**
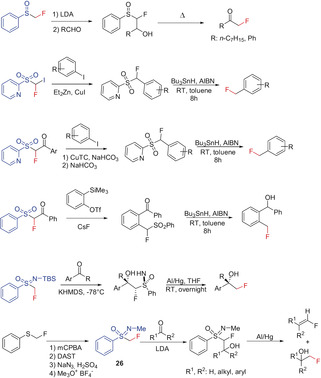
Fluoromethylation with fluoromethyl phenyl sulfoxide and fluoromethyl phenyl sulfone derivatives.

A carbanion with a fluorine atom directly bonded to the negatively charged carbon atom can also be stabilized by a sulfoxide group. Deprotonation of fluoromethyl phenyl sulfoxide at the methylene group with LDA at −78 °C results in the formation of a carbanion, which is moderately stable at low temperatures. Reaction with aldehydes followed by pyrolysis generates the corresponding fluoromethyl ketones in moderate yields (Scheme 15).^63^ An aromatic fluoromethylation with an α‐fluoro‐β‐keto phenyl sulfone, acting as a soft nucleophile, has been reported by Hu and co‐workers.^62^ The three‐step synthesis involves the addition to a benzyne generated in situ, followed by the reduction of the keto group and the reductive cleavage (Na/Hg) of the sulfonyl moiety.^62^ In addition to the frequently used fluoromethyl phenyl sulfone **22**, derivatives of **22**, described by Hu and co‐workers in 2012–2014, with substituents at the fluoromethyl carbon atom or the analogous fluoromethyl TBS‐sulfoxinimine have also been used to prepare the corresponding fluoromethyl products (Scheme 15).^15^, 27f, 64 Some of the syntheses involve transition‐metal‐mediated C−C coupling reactions.^15^, 27f, 64a, 64b Finch and co‐workers described in 1988 the use of sulfoximine **26** as a nucleophilic source for the fluoromethyl group. Its reaction with aldehydes and ketones in the presence of a base proceeds with addition to the C=O bond yielding the corresponding β‐fluorosulfonyl alcohols. The reductive cleavage of the sulfonyl substituent with aluminum amalgam produces the respective fluorine‐substituted olefins together with the fluoromethyl alcohols. In the case of R^1^=H and R^2^=4‐MeOC_6_H_4_, the fluoromethyl alcohol is obtained in 57 % yield when sodium amalgam is used (Scheme 15).^65^

#### 3.1.3 Fluorobis(phenylsulfonyl)methane

**Scheme 16:**
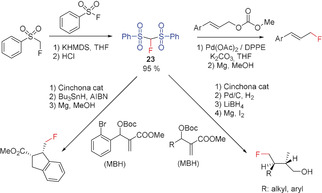
Reactions of FBSM (**23**) with MBH carbonates.

Since the discovery of fluorobis(phenylsulfonyl)methane (FBSM, **23**) in 2006 by Hu/Shibata and co‐workers and its suitability as a fluoromethylating agent, a number of fluoromethylation reactions, including transition‐metal‐mediated cross‐couplings, have been performed.^17^, 28i, 66 The synthesis of **23** has also been improved. A convenient method for the preparation of **23** is the reaction of fluoromethyl phenyl sulfone (**22**) with phenylsulfonyl fluoride.5c Hu and Prakash reported that FBSM acts as a nucleophilic fluoromethylating reagent and undergoes addition reactions with epoxides,66a aziridines,^62^ α,β‐unsaturated ketones,^62^, ^67^ alkynyl ketones,^62^ and benzynes.^62^ Shibata and Prakash found **22** to be an effective reagent in the palladium‐catalyzed enantioselective fluoromethylation of allylic acetates, imines, and α,β‐unsaturated ketones and esters.5b, 67 Further, the fluoromethylation of alcohols, alkyl halides, and α,β‐unsaturated ketones with **23** (using a cinchona alkaloid derived catalyst) has been reported.7a, 67b Using an in situ formed iminium compound as the catalyst, Wang et al. reported an enantioselective addition of **23** to enals.^68^ In the last ten years, some research groups have described the reaction of FBMS with aliphatic aldehydes resulting in enantioselective fluoromethylation in the β‐position,^69^ as well as the addition of FBMS to MBH carbonates or acetates yielding the products of an enantioselective asymmetric allylic alkylation (Scheme 16).^70^ Gouverneur and co‐workers showed that the palladium‐catalyzed allylic alkylation reaction of **23** with Morita–Baylis–Hillmann (MBH) carbonates (allyl carbonates) proceeds with high regioselectivity.^71^ The addition to alkyl and benzyl halides also proceeds with high yields, as shown by Olah and co‐workers.^72^ The fluoromethyl group is finally formed after reductive cleavage of the sulfonyl substituents with Mg in MeOH (Scheme 16).70c, 71, 72, 73 Instead of the palladium catalyst, the combination of a cinchona alkaloid and FeCl_2_ or a cinchona‐catalyzed Mannich‐type reaction can be used for enantioselective monofluoromethylation (Shibata and co‐workers).^74^ Furthermore, the addition of **23** to carbonyl compounds,69a α,β‐unsaturated carbonyl compounds,^75^ and functionalized alkynes^76^ as well as the enantioselective synthesis of tertiary allylic fluorides by iridium‐catalyzed allylic fluoromethylation with **23** have been described by the groups of Hu, Vesely, and Hartwig.^77^ Reductive cleavage of the sulfonyl substituents to yield the corresponding fluoromethyl derivatives, as in the other examples discussed above, was not reported.

**Scheme 17:**
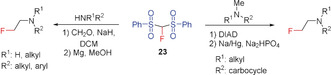
Synthesis of β‐fluoro ethylamines using **23**.

The reaction of **23** with MBH carbonates (Toru and Tan) proceeds with high enantio‐ and diastereoselectivity and yields alcohols with a fluoromethyl group in γ‐position to the OH group after workup.^74^, ^78^ The introduction of a fluoromethyl group in Ibuprofen by using **23** in place of the methyl group results in an increase in its inhibitory activity.^79^ The reaction of secondary amines with formaldehyde in the presence of FBSM (Prakash et al., 2013) opens up a general and straightforward synthetic route to β‐fluoro ethylamines.^80^ Hu and co‐workers reported in the same year that starting from tertiary amines, further β‐fluoro ethylamines can be prepared by C−C coupling using **23** and diisopropyl azodicarboxylate (DIAD) as the coupling reagent (Scheme 17).77b

**Scheme 18:**
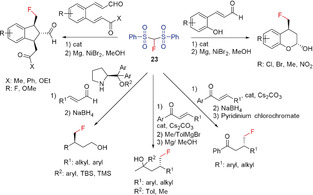
Reaction of FBSM (**23**) with enals and enones.

In 2014, Ramos and Yang extended the addition reaction of FBSM to enals, providing an enantioselective synthesis for fluoroindane and fluorochromanol derivates (Scheme 18).^81^

**Scheme 19:**
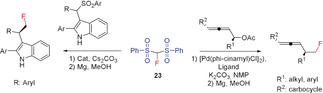
Synthesis of fluoromethyl‐containing aryl‐substituted indoles and allenes with **23**.

Shibata and co‐workers reported an efficient method for preparing C2‐aryl indoles with a fluoromethyl group at the alkyl side chain starting from the corresponding aryl sulfonyl derivatives and replacing the SO_2_Ar substituent by CH_2_F, by utilizing **23** in the presence of a chiral phase transfer catalyst.^82^ Furthermore, the acetate group of allenyl acetates can be replaced with a CH_2_F group by employing **23** (Ma and Lu), yielding the corresponding fluoromethyl allenes (Scheme 19).^83^ FBSM is also the key reagent of a highly selective two‐step synthesis of functionalized monofluoromethylated allenes, reported by Shibata and co‐workers.^84^ In the first step, 2‐bromo‐1,3‐dienes react with FBSM in a palladium‐catalyzed nucleophilic substitution that selectively introduces the fluorobis(phenylsulfonyl)methyl group directly bonded to the allene skeleton. The following reductive desulfonation (Mg, MeOH) gives the fluoromethyl allenes in excellent (81–83 %) yields.^84^

**Scheme 20:**
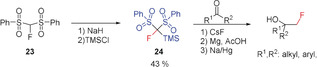
Synthesis of **24** and its use for the preparation of α‐fluoromethyl alcohols.

An efficient synthesis of α‐fluoromethyl alcohols has been reported by Prakash and Olah in 2012, using the related trimethylsilyl derivative **24**. This reagent contains a SiMe_3_ group in place of the hydrogen atom of FBSM and is readily prepared from **23** by deprotonation with NaH and subsequent silylation with Me_3_SiCl (Scheme 20).^85^

#### 3.1.4 2‐Fluoro‐1,3‐benzothiole‐1,1,3,3‐tetraoxide

**Scheme 21:**
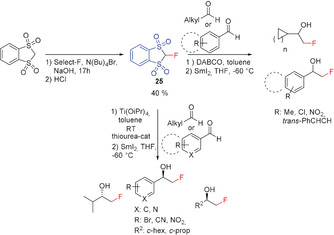
Synthesis of **25** and its reaction with aldehydes.

The cyclic version (FBDT) **25** of FBSM was reported in 2010 by Shibata and co‐workers.^86^ Reagent **25** is prepared starting from the corresponding methylene‐bridged derivative by fluorination with Selectfluor and formed as a colorless solid. FBDT adds efficiently to the C=O group of a variety of aldehydes yielding the corresponding α‐fluoromethyl alcohols after workup. The addition is complete within 24 h. In the case of α,β‐unsaturated aldehydes, 1,2‐addition competes with 1,4‐addition, and the selectivity is strongly dependent on the base used (DABCO or pyrrolidone).^86^ In the presence of bifunctional cinchona alkaloid derived thiourea titanium complexes, the reaction of **25** with aldehydes becomes enantioselective (32–96 % *ee*) and yields the fluoromethyl alcohols in 73–91 % yield (Scheme 21).^87^ The structure of FBDT (**25**) has been determined in the solid state by single‐crystal X‐ray diffraction.^86^

### 3.2 Phosphorus‐Containing Fluoromethyl Precursors

**Scheme 22:**
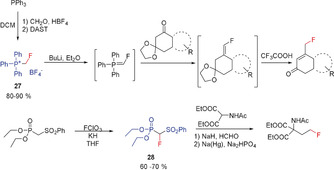
Fluoromethylation reactions with the phosphorus reagents **27** and **28**.

Fluoromethyl triphenylphosphonium tetrafluoroborate (**27**) has been utilized as a precursor to generate the corresponding fluoromethylidene phosphonium ylide, which has been employed in Wittig‐type reactions for the synthesis of fluoroalkenes. In the case of a special ketone (Scheme 22), a subsequent proton shift catalyzed by trifluoroacetic acid results in the formation of a fluoromethyl derivative (Bohlmann and co‐workers, 1995).9c, 88 The structure of the fluoromethyl triphenylphosphonium cation salt in the solid state as its iodide salt has been determined by single‐crystal X‐ray diffraction.^8^

The α‐fluoromethyl phosphonate **28** displays increased acidity for the proton in α‐position, supported by the electron‐withdrawing sulfonyl group. Its reaction with formaldehyde (Takeuchi and co‐workers, 1987) results in the formation of the corresponding sulfonyl‐substituted fluoroalkene, which can be converted with the anion of diethyl acetamido malonate into the corresponding fluoromethyl derivative after reductive elimination of the sulfonyl group (Scheme 22).^89^

## 4 Conclusion

The unique properties of organic molecules containing a fluoromethyl (CH_2_F) group and their use in various fields of pharmacy and medicine has resulted in a high demand for reagents that are capable of selectively introducing a CH_2_F group. In recent decades, great efforts have been made in the development of fluoromethylating reagents and several new reagents have been prepared and used. Most of the reagents are based on fluorohalomethanes and, more specifically, fluorochloromethane, or derivatives thereof. The main synthetic strategies are the introduction of a suitable leaving group in place of the halogen (Cl, Br, I), or the introduction of electron‐withdrawing substituents at the carbon atom bonded to fluorine. In the former case, the CH_2_F group is transferred as the electrophile. The alkylation strengths of the reagents differ and can be fine‐tuned by the nature of the respective leaving group. In the latter case, electron‐withdrawing substituents (SO_2_Ar, PhCH_2_OC(O), PhS(O)NTBS) stabilize a negative charge at the carbon atom bonded to fluorine, and CH_2_F is introduced as a nucleophile; the reagent can thus be considered as a replacement for the unstable and very sensitive FCH_2_Li. Over the last decade, particular attention has been paid to reagents that are able to transfer the CH_2_F group by a radical pathway. The strategy behind this approach was again the introduction of suitable substituents at the carbon atom bonded to fluorine that favor radical formation. Despite the great progress that has been made, most of the reagents are effective in transferring CH_2_F only to heteroatoms (nitrogen, oxygen, sulfur). The transfer of CH_2_F with concurrent C−C bond formation is less effective, and the development of readily available fluoromethylating reagents capable of achieving this goal still remains a challenge for organofluorine chemists.

## Conflict of interest

The authors declare no conflict of interest.

## Biographical Information

*Marco Reichel received his M. Sc. degree in chemistry from Ludwig‐Maximilian University. He is currently completing his PhD thesis under the supervision of Prof. K. Karaghiosoff. His research is focused on the development of new selective fluoromethylating agents and on studying and understanding the effect of the monofluoromethyl unit on energetic materials*.

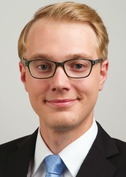

## Biographical Information

*Konstantin Karaghiosoff is professor for inorganic chemistry at Ludwig‐Maximilian University and subgroup leader at the chair of Prof. T. M. Klapötke. He is a member of the GDCh and European Editor of “Phosphorus*, *Sulfur, Silicon and Related Elements”. His research interests, in addition to multinuclear NMR spectroscopy and the analysis of high‐order NMR spectra, are focused on phosphorus compounds for possible application in OLEDs, as well as phosphonates and organofluorine compounds for pharmaceutical or energetic use*.